# Improved production of *Bacillus subtilis* cholesterol oxidase by optimization of process parameters using response surface methodology

**DOI:** 10.1186/s43141-023-00576-9

**Published:** 2023-11-24

**Authors:** Walid A. Lotfy, Hala M. Badawy, Khaled M. Ghanem, Samy A. El-Aassar

**Affiliations:** 1 Department of Microbiology, Faculty of Dentistry, Pharos University in Alexandria, Alexandria, Egypt; 2 Department of Botany and Microbiology, Faculty of Science, Alexandria University, Alexandria, Egypt

**Keywords:** *Bacillus subtilis*, Box–Behnken design, Cholesterol oxidase, Optimization, Plackett–Burman design

## Abstract

**Background:**

Cholesterol oxidase has numerous biomedical and industrial applications. In the current study, a new bacterial strain was isolated from sewage and was selected for its high potency for cholesterol degradation (%) and production of high cholesterol oxidase activity (U/OD_600_).

**Results:**

Based on the sequence of 16S rRNA gene, the bacterium was identified as *Bacillus subtilis*. The fermentation conditions affecting cholesterol degradation (%) and the activity of cholesterol oxidase (U/OD_600_) of *B. subtilis* were optimized through fractional factorial design (FFD) and response surface methodology (RSM). According to this sequential optimization approach, 80.152% cholesterol degradation was achieved by setting the concentrations of cholesterol, inoculum size, and magnesium sulphate at 0.05 g/l, 6%, and 0.05 g/l, respectively. Moreover, 85.461 U of cholesterol oxidase/OD_600_ were attained by adjusting the fermentation conditions at initial pH, 6; volume of the fermentation medium, 15 ml/flask; and concentration of cholesterol, 0.05 g/l. The optimization process improved cholesterol degradation (%) and the activity of cholesterol oxidase (U/OD_600_) by 139% and 154%, respectively. No cholesterol was detected in the spectroscopic analysis of the optimized fermented medium via gas chromatography-mass spectroscopy (GC–MS).

**Conclusion:**

The current study provides principal information for the development of efficient production of cholesterol oxidase by *B. subtilis* that could be used in various applications.

## Background

Cholesterol (5-cholesten-3-ol) is a major lipid molecule in the cell membrane of eukaryotic cells and an essential precursor in the biosynthesis of other lipids [1]. Cholesterol oxidase is involved in the degradation of cholesterol to 4-cholesten-3-one [2]. Cholesterol oxidase has various industrial and medicinal applications such as the determination of serum and food cholesterol levels and synthesis of intermediates for the production of steroid drugs, prevention of skin keratinization, treatment of liver diseases, and control of obesity [2, 3]. Bacteria use cholesterol oxidase for oxidation of cholesterol in host macrophages during infection or for the decomposition of cholesterol to obtain their carbon source [3]. Cholesterol oxidase has been isolated from several Gram-positive and Gram-negative bacteria such as *Brevibacterium* [4], *Pseudomonas* [5], *Mycobacterium* [6], *Nocardia* [7], *Arthrobacter* [8], *Rhodococcus* [9], *Burkholderia* [10], *Streptomyces* [11], *Cellulomonas* [12], *Chromobacterium* [13], and *Schizophyllum* [14]. However, *Streptomyces* is the most widely used microbial source for the industrial production of cholesterol oxidase.

There are few reports in the literature focusing on the production of cholesterol oxidase from the Gram-positive endospore-forming *Bacillus* spp. [15–22]. Two cholesterol oxidases were purified from *Bacillus* sp. SFF34 that was isolated from Korean traditional fermented flatfish [18]. Production of cholesterol oxidase from two strains of *B*. *cereus* viz., KAVK4 and KAVK5, has been also optimized [17]. Optimization of the fermentation medium enhanced the productivity of cholesterol oxidase from *B*. *cereus* KAVK5 affording a consistent conversion of cholesterol into 4-cholesten-3-one [20]. The bioconversion of cholesterol into 4-cholesten-3-one has been also performed using *B*. *subtilis* 168 cloned with cholesterol oxidase genes from *Mycobacterium neoaurum* JC-12 [19]. The production of cholesterol oxidase from *B*. *pumilus* was optimized using classical methods [21] and via Plackett–Burman and Box–Behnken statistical approach [15]. The traditional one variable at a time (OVAT) and the Plackett–Burman design were also used to optimize the production of cholesterol oxidase from *B*. *cytotoxicus* [22]. *Bacillus* sp. that was isolated from tiger fecal samples and biochemically identified as *B*. *subtilis* was capable of producing cholesterol oxidase [16].

Screening and predicting the levels of optimal variables using a mathematical model through fractional factorial design (FFD) and response surface methodology (RSM) have been reported in various biotechnological applications [23–28]; however, these techniques have not been applied to the synthesis of cholesterol oxidase by *B. subtilis*. In the present work, FFD and RSM were implemented to enhance the cholesterol oxidase synthesis by *B. subtilis*. Optimization of the process parameters affecting the production of cholesterol oxidase by *B. subtilis* was thoroughly considered. Moreover, this investigation will be valuable for the development of efficient large-scale production of cholesterol oxidase by *B. subtilis*.

## Methods

### Media and chemicals

The following media were used throughout the current study: nutrient broth (NB) composed of (g/l) peptone, 5; beef extract, 1.5; yeast extract, 1.5; sodium chloride, 5, pH 7.4 ± 0.2; and modified minimal salt (MMS) medium composed of (g/l) K_2_HPO_4_, 0.25; NaCl, 1; MgSO_4_, 0.025; FeSO_4_, 0.01; KNO_3_, 1 and cholesterol, 1. Cholesterol was used as the sole carbon source and was prepared separately by dissolving 1 g of cholesterol in 20 ml tween 80 previously heated to 90 °C for 5 min, then added to medium components. All media were prepared and sterilized according to the instructions of the manufacturer. Sterilization was carried out by autoclaving (SJ-FW, Shin Jin Engineering, Korea), and media were prepared by the addition of 20 g/l agar to the liquid medium. Croma-test cholesterol determination kit was purchased from Linear Chemicals, Barcelona, Spain. All chemicals used in the present work were of analytical grade (HiMedia Laboratories, India).

### Microorganism

The bacterium used throughout this work was isolated from the sewage of Edko 9 (geographic coordinates 31.299146, 30.308738), Al-Behira governorate, Egypt. The bacterial isolate was molecularly identified by sequencing 16S rRNA gene. The genomic DNA was isolated and purified according to a protocol stated by Sambrook et al. [29]. Polymerase chain reaction (PCR) was used in the amplification of the full length of 16S rRNA gene (1500 bp) of the bacterial isolate. A universal 16S rRNA primer set F8-27 (5′-AGAGTTTGATCCTGGCTCAG-3′) and R-1510–1492 (5′GGTTACCTGTTACGACTT-3′) was used in the current study [30]. The reaction mixture included the following: 5 µl of genomic DNA (30 ng), forward and reverse primer (2 µl), 25 µl of 2 × TAQ Green master mix (Thermo Fisher Scientific Co., USA) and 16 µl of nuclease free water. PCR conditions were applied as follows: initial denaturation at 95 °C for 5 min, 30 cycles each cycle (denaturation 94°C, 45 s; annealing 55 °C, 45 s; and extension 72 °C, 1.5 min, and a final extension of 72 °C for 10 min). After termination of PCR, aliquots of each PCR reaction were set to run on 1% agarose gel electrophoresis along with DNA ladder to check the PCR product. The PCR product of 16S rRNA gene was purified using PCR clean-up Kit SV Wizard (Promega Co., USA) according to the instructions of the manufacturer. The purified PCR product was sequenced along with the aforementioned universal primer set. The obtained nucleotide sequence of the 16S rRNA gene was processed to select the clean nucleotide sequence with sharp non-overlapped peaks using Bio Edit version 7.0.5.3 software. The processed sequence of nucleotides was analyzed by BLASTN (Basic Local Alignment Search Tool, Nucleotide) of the National Centre for Biotechnology Information (NCBI) to determine the hits of subject sequences deposited in the international nucleotide databases giving the best matching with the query sequence. Based on the multiple sequence alignment result, a phylogenetic tree was constructed via CLC Sequence Viewer 6.8 software to determine the taxonomic correlation with closely related bacteria to the bacterial isolate.

### Seed inoculum

An Erlenmeyer flask of volume 100 ml containing 25 ml NB was used for growing the isolated *B. subtilis* strain at 30 °C under shaking at 110 rpm until the optical density (OD) reaches 1 at 600 nm. The OD was measured using spectrophotometer (T80 + UV/VIS, PG Instruments LTD, UK). An inoculum size (%) from the broth culture was used as the seed bacterial inoculum as specified under each experiment.

#### Cholesterol assay

Culture supernatant fluid was obtained by centrifugation of the culture broth for 20 min at 10,000 × g and 4 °C, and then residual cholesterol was determined using a Croma-test kit. An aliquot of 10 µl was taken from the culture supernatant mixed with 1 ml from the Croma-test reagent for 10 min then incubated at 37 °C for 5 min. The pink color which is stable for at least 30 min was measured at 500 nm against the reagent blank.

1 $$\mathrm{Cholesterol}\;\mathrm{degradation}\left(\%\right)=\frac{\mathrm{Initial}\;\mathrm{cholesterol}\;\mathrm{concentration}-\mathrm{Remanining}\;\mathrm{cholesterol}\;\mathrm{concentration}}{\mathrm{Initial}\;\mathrm{cholesterol}\;\mathrm{concentration}}\times100$$

### Enzyme assay

The activity of cholesterol oxidase was measured according to the method described by Richmond [31] which is based on the conversion of cholesterol into 4-cholestene-3-one. Crude enzyme preparation was obtained by cooling centrifugation of the broth bacterial culture at 5000 × g and 4 °C [32]. The reaction mixture contained 50 µl of the crude enzyme, 3 ml buffered Triton X-100 pH 7.0, and 50 µl of 12 mM of cholesterol dissolved in isopropanol. The contents of the reaction mixture were mixed by inversion, and the absorbance was measured at 240 nm. The concentration of 4-colesten-3-one was calculated from a previously prepared standard curve. One unit of cholesterol oxidase has been defined as the amount of enzyme required to produce 1.0 µmol of 4-cholesten-3-one per min at pH 7.0 and 37 °C. The enzymatic activity of cholesterol oxidase was expressed in U/OD_600_ by dividing the number of enzyme units per ml by the OD of bacterial growth at 600 nm.

### Time course of cholesterol oxidase production

Growth and cholesterol oxidase activity of *B. subtilis* were monitored at different time intervals during incubation for 48 h (New Brunswick Scientific Edison, USA). Bacterial growth on MMS medium was estimated by measuring the OD at 600 nm.

### Optimization of cholesterol degradation and cholesterol oxidase activity

Optimization of cholesterol degradation (%) and the activity of cholesterol oxidase (U/OD_600_) were performed through two experimental design steps. In the first step, a FFD, the Plackett–Burman design [33], was applied to assess the significance of medium constituents and fermentation parameters on cholesterol degradation (%) and the activity of cholesterol oxidase (U/OD_600_). In the second phase, levels of variables that significantly influenced cholesterol degradation (%) and the activity of cholesterol oxidase (U/OD_600_) were further investigated using a RSM design, the Box–Behnken design [34]. The basal medium composition of the Plackett–Burman design was formulated based on some preliminary studies.

### Plackett–Burman design

The Plackett–Burman design is a two-level FFD (− 1 and + 1) that locates significant variables for the production by screening “*n*” variables in “*n* + 1” experiments. All nine factors chosen in the present investigation were tested at these two levels, based on the Plackett–Burman matrix design. After incubation for 48 h at 30 °C under shaking at 110 rpm, the main effect was calculated basically as a difference between the average measurements of each variable made at a high level (+ 1) and a low level (− 1). According to the following equation:

2 $$\mathrm{Exi }= \frac{\sum Pi+ - \sum Pi-}{N}$$

where Exi is the variable main effect, Pi + and Pi − are cholesterol degradation (%) or cholesterol oxidase activity (U/OD_600_) in trials where the independent variable (Xi) was present in high and low concentrations, respectively, and *N* is the number of trials divided by 2. The main effect figure with a positive sign indicates that the high concentration of this variable is nearer to the optimum, and a negative sign indicates that the low concentration of this variable is nearer to the optimum. Statistica 10.0 software was used to determine the main effect and the significance of each variable.

### Box–Behnken design

In the second phase of optimizing cholesterol degradation (%) and cholesterol oxidase activity (U/OD_600_), the Box–Behnken design was applied. In this model, the most three significant independent variables, coded *X*1, *X*2, and *X*3, were included in the matrix design and each factor was examined at three different levels, low ( −), high ( +) and central or basal (0). Fifteen combinations were constructed, and their observations were determined after incubation for 48 h at 30 °C under shaking at 110 rpm. The observations were fitted to the following second-order polynomial model:

3 $$\mathrm{Y }=\mathrm{ b}0\;+\;\mathrm{ b}1\mathrm{X}1\;+\;\mathrm{ b}2\mathrm{X}2\;+\;\mathrm{ b}3\mathrm{X}3\;+\;\mathrm{ b}12\mathrm{X}1\mathrm{X}2\;+\;\mathrm{ b}13\mathrm{X}1\mathrm{X}3\;+\;\mathrm{b}23\mathrm{X}2\mathrm{X}3\;+\;\mathrm{ b}11\mathrm{X}{1}^{2}\;+\;\mathrm{ b}22\mathrm{X}{2}^{2}\;+\;\mathrm{ b}33\mathrm{X}{3}^{2}$$

where *Y* is the dependent variable cholesterol degradation or cholesterol oxidase activity; *X*1, *X*2, and *X*3 are the independent variables; *b*0 is the regression coefficient at center point; *b*1, *b*2, and *b*3 are linear coefficients; *b*12, *b*13, and *b*23 are second-order interaction coefficients; and *b*11, *b*22, and *b*33 are quadratic coefficients. The regression analysis, the values of the coefficients, and the optimum concentrations were determined using Statistica 10.0 software.

### Determination of biodegradation end products

The bacterial culture was grown under the optimized conditions described under the Results section, collected, and centrifuged at 5000 × g at 4 °C, and the supernatant was filtered using a 0.45-µm nitrocellulose filter. The solution was injected into GC–MS (Thermo Agilent Technologies—7890A, USA) using helium as carrier gas in Trace Gold System Qualification Column (TG-SQC). Analysis of samples was applied on temperature program as follows: initial temperature 50 °C for 1 min, then increased to 250 °C for 5 min, finally increased to 290 °C for 2 min. Samples were injected in split mode constant flow 1.5 ml/min. Mass spectral range was set as 40–1000 Hz, mass transfer line temperature was 300 °C, and ion source temperature was 300 °C. Identification of components was performed by the use of computer search on Wiley Spectral Libraries database.

### Statistical analysis

Each experiment was carried out three times in triplicate; the mean and standard deviation were calculated using Microsoft Excel 2010.

## Results

### Time course of cholesterol degradation and cholesterol oxidase synthesis by the bacterial isolate

**Fig. 1:**
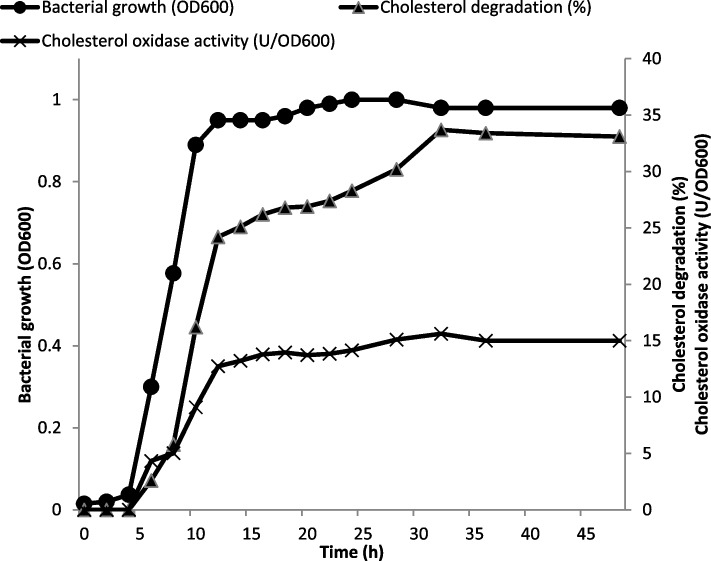
Time course of growth, cholesterol degradation (%), and cholesterol oxidase activity (U/OD_600_) of the new bacterial isolate

Cholesterol degradation and cholesterol oxidase activity of the bacterial isolate were monitored at different time intervals for 48 h. As shown in Fig. 1, the log phase of the bacterial isolate started at 5 h of the incubation, while the stationary phase started at 12 h of the incubation and the growth reached a maximum after 24 h. The activity of cholesterol oxidase (U/OD_600_) increased significantly between 6 and 12 h. The highest activity of cholesterol oxidase (15.61 U/OD_600_) was recorded at 32 h of incubation. On the other hand, a steady rise in the percentage of cholesterol degradation over time was obvious. However, the degradation percentage of cholesterol did not increase further after 32 h, achieving its maximum value of 33.7%. It was also concomitant with the activity of cholesterol oxidase (U/OD_600_).

**Fig. 2:**
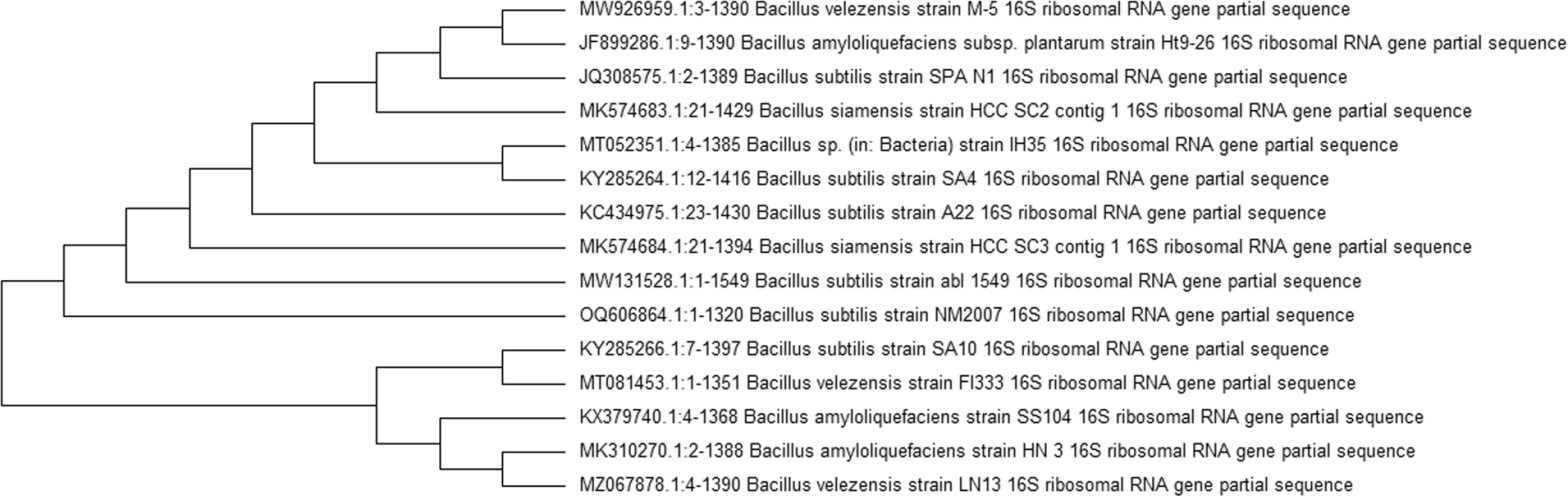
Phylogenetic tree showing the query strain in relation to other *Bacillus* species

The bacterial isolate was identified via sequencing of the 16S rRNA gene approach. BLASTN analysis, multiple sequence alignments, and phylogenetic tree (Fig. 2) revealed that 16S rRNA nucleotide sequence of the bacterium exhibited 100% identity with 16S rRNA sequence of *B*. *subtilis*. The sequence has been deposited in the GenBank under accession number MW131528.1, and the strain was designated as *B. subtilis* abl 1549.

### Evaluating the significance of fermentation conditions affecting cholesterol degradation (%) and cholesterol oxidase activity (U/OD_600_) of *B*. *subtilis*

The Plackett–Burman design was implemented for screening the most significant fermentation factors governing cholesterol degradation (%) and cholesterol oxidase activity (U/OD_600_) of *B. subtilis*. Nine factors were included in the design with 13 different combinations; cholesterol degradation (%) and cholesterol oxidase activity (U/OD_600_) were observed (Table 1).

**Table 1:** A randomized Plackett–Burman design applied on the degradation of cholesterol (%) and the activity of cholesterol oxidase (U/OD_600_) produced by *B. subtilis*

Trial	Independent variable									Dependent variable	
	**C (g/l)**	**PP (g/l)**	**PN (g/l)**	**S (g/l)**	**M (g/l)**	**F (g/l)**	**V (ml/flask)**	**I (%)**	**pH**	**Cholesterol degradation (%)**	**Cholesterol oxidase activity (U/OD**_**600**_**)**
1	6.0	0.125	1.5	0.5	0.0125	0.005	75	6.0	8	17.0	32.72
2	4.0	0.250	1.0	1.0	0.025	0.01	50.0	4.0	7.0	33.8	28.28
3	6.0	0.375	0.5	1.5	0.0125	0.005	25	6.0	8	15.4	67.48
4	2.0	0.375	0.5	0.5	0.0125	0.015	75	6.0	6	44.8	37.93
5	2.0	0.375	1.5	0.5	0.0375	0.005	25	2.0	8	35.0	27.32
6	2.0	0.125	0.5	1.5	0.0375	0.015	25	6.0	8	49.0	32.57
7	6.0	0.125	1.5	1.5	0.0125	0.015	25	2.0	6	15.3	68.03
8	2.0	0.375	1.5	1.5	0.0125	0.015	75	2.0	8	21.4	29.82
9	6.0	0.375	0.5	1.5	0.0375	0.005	75	2.0	6	19.2	66.45
10	6.0	0.375	1.5	0.5	0.0375	0.015	25	6.0	6	26.4	60.43
11	2.0	0.125	1.5	1.5	0.0375	0.005	75	6.0	6	41.7	33.18
12	6.0	0.125	0.5	0.5	0.0375	0.015	75	2.0	8	20.9	15.27
13	2.0	0.125	0.5	0.5	0.0125	0.005	25	2.0	6	36.1	61.87

*Abbreviations*: *C* cholesterol concentration, *PP* potassium phosphate, *PN* potassium nitrate, *S* sodium chloride, *M* magnesium sulphate, *F* ferrous sulphate, *V* volume of the media, *I* inoculum size

**Fig. 3:**
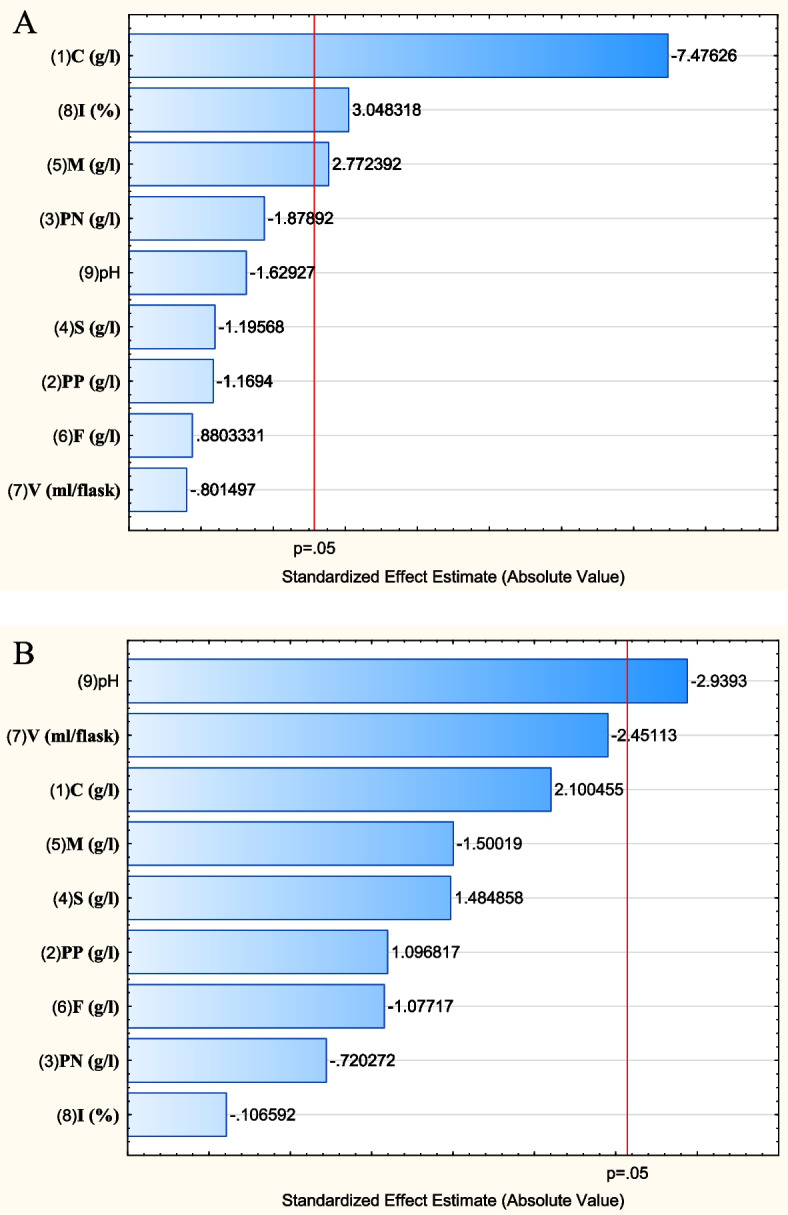
The main effect of fermentation factors on *B. subtilis* cholesterol degradation (%) A and cholesterol oxidase activity (U/OD_600_) B. *Abbreviations*: *C *cholesterol concentration, *PP *potassium phosphate, *PN *potassium nitrate, *S *sodium chloride, *M *magnesium sulphate, *F *ferrous sulphate, *V *volume of the fermentation media, *I *inoculum size

The results represented in Table 2 and Fig. 3A indicate that increasing the level of inoculum size (%), magnesium sulphate (g/l), and ferrous sulphate (g/l) is advantageous for cholesterol degradation (%) by *B. subtilis*. On the contrary, the high level of cholesterol concentration (g/l), potassium nitrate (g/l), pH, sodium chloride (g/l), potassium phosphate (g/l), and volume of the media (ml/flask) negatively affected cholesterol degradation (%) by *B. subtilis*. Moreover, according to the calculated *t*-values, the results suggest that cholesterol concentration (g/l), inoculum size (%), and magnesium sulphate (g/l) were the most significant variables that influence cholesterol degradation (%) by *B. subtilis.*

**Table 2:** Statistical analysis of the Plackett–Burman experimental results

Variable	Cholesterol degradation (%)				Cholesterol oxidase activity (U/OD_600_)			
	**Coefficient**	**Main effect**	***t*-value**	***p*-value**	**Coefficient**	**Main effect**	***t*-value**	***p*-value**
**C (g/l)**	− 9.48333	− 18.966	− 7.47626	0.000676*	7.3075	14.6150	2.10046	0.089701
**PP (g/l)**	− 1.48333	− 2.9667	− 1.16940	0.294952	3.8158	7.6317	1.09682	0.322714
**PN (g/l)**	− 2.38333	− 4.7667	− 1.87892	0.119042	− 2.5058	− 5.0117	− 0.7202	0.503612
**S (g/l)**	− 1.51667	− 3.0333	− 1.19568	0.285431	5.1658	10.3317	1.48486	0.197710
**M (g/l)**	3.51667	7.0333	2.77239	0.039254*	− 5.2192	− 10.438	− 1.5001	0.193857
**F (g/l)**	1.11667	2.2333	0.88033	0.418975	− 3.7475	− 7.4950	− 1.0771	0.330606
**V (ml/flask)**	− 1.01667	− 2.0333	− 0.80150	0.459223	− 8.5275	− 17.055	− 2.4511	0.057856
**I (%)**	3.86667	7.7333	3.04832	0.028479*	− 0.3708	− 0.7417	− 0.1065	0.919258
**pH**	− 2.06667	− 4.1333	− 1.62927	0.164184	− 10.2258	− 20.451	− 2.9393	0.032283*

*Abbreviations*: *C* cholesterol concentration, *PP* potassium phosphate, *PN* potassium nitrate, *S* sodium chloride, *M* magnesium sulphate, *F* ferrous sulphate, *V* volume of the media, *I* inoculum size

^*^Significant (*p*-value ≤ 0.05)

The magnitude of each of the nine independent variables on the activity of *B. subtilis* cholesterol oxidase (U/OD_600_) is shown in Table 2 and Fig. 3B. The results revealed that high levels of cholesterol concentration (g/l), sodium chloride (g/l), and potassium phosphate (g/l) were advantageous for the activity of cholesterol oxidase (U/OD_600_) produced by *B. subtilis*. On the other hand, the enzyme activity (U/OD_600_) was negatively affected by high initial pH, higher volume of fermentation medium (ml/flask), and the presence of high concentrations of magnesium sulphate (g/l), ferrous sulphate (g/l), potassium nitrate (g/l), and inoculum size (%) in the fermentation medium. Based on the statistical analysis performed (Table 2), initial pH, volume of fermentation medium (ml/flask), and cholesterol concentration (g/l) were considered significant variables with respect to the activity of *B. subtilis* cholesterol oxidase (U/OD_600_).

The results virtually demonstrated an imminent optimized medium composition for maximum degradation of cholesterol (%) by *B. subtilis* as follows: cholesterol concentration, 2 g/l; potassium phosphate, 0.125 g/l; potassium nitrate, 0.5 g/l; sodium chloride, 0.5 g/l; magnesium sulphate, 0.0375g/l; ferrous sulphate, 0.015g/l; volume of the medium, 25 ml; inoculum size, 6%; and initial pH of fermentation medium, 6. On the other hand, a proximate optimized medium for maximum activity of cholesterol oxidase production by *B. subtilis* is recommended to be as follows: cholesterol concentration, 6 g/l; potassium phosphate, 0.375 g/l; potassium nitrate, 0.5 g/l; sodium chloride, 1.5 g/l; magnesium sulphate, 0.0125 g/l; ferrous sulphate, 0.005 g/l; volume of the medium, 25 ml; inoculum size, 2%; and initial pH of fermentation medium, 6.

### Optimization of fermentation conditions affecting cholesterol degradation (%) and the activity of cholesterol oxidase (U/OD_600_) produced by *B*. *subtilis*

Based on the Plackett–Burman experimental results, the significant factors affecting cholesterol degradation (%) by *B. subtilis* and the activity of *B. subtilis* cholesterol oxidase (U/OD_600_) were selected, and their levels were further investigated using the Box–Behnken design. For cholesterol degradation (%), the interactions of cholesterol concentration (g/l) with the inoculum size (%) and the concentration of magnesium sulphate (g/l) were examined. Whereas, the activity of cholesterol oxidase (U/OD_600_) produced by *B. subtilis* was further studied using the most effective key factors viz, initial pH, volume of the medium (ml/flask), and cholesterol concentration (g/l) as independent variables. In both cases, each independent variable was examined at three different levels in 15 trials as shown in Table 3, while the other factors were set at their basal levels. The basal condition for cholesterol degradation (%) by *B. subtilis* was as follows: cholesterol concentration, 2 g/l; potassium phosphate, 0.125 g/l; potassium nitrate, 0.5 g/l; sodium chloride, 0.5 g/l; magnesium sulphate, 0.0375 g/l; ferrous sulphate, 0.015 g/l; volume of the medium, 25 ml; inoculum size, 6%; and initial pH, 6. On the other hand, the basal condition for the activity (U/OD_600_) of *B. subtilis* cholesterol oxidase was as follows: cholesterol concentration, 6 g/l; potassium phosphate, 0.375 g/l; potassium nitrate, 0.5 g/l; sodium chloride, 1.5 g/l; magnesium sulphate, 0.0125 g/l; ferrous sulphate, 0.005 g/l; volume of the medium, 25 ml; inoculum size, 2%; and initial pH of fermentation medium, 6.

**Table 3:** Concentrations of the key variables examined in the randomized Box–Behnken experiment and the observed responses of cholesterol degradation (%) and cholesterol oxidase activity (U/OD_600_) of *B. subtilis*

Trial	Cholesterol degradation				Cholesterol oxidase activity			
	**Cholesterol (g/l)**	**Inoculum size (%)**	**Magnesium sulphate (g/l)**	**Cholesterol degradation (%)**	**pH**	**Volume of the medium (ml/flask)**	**Cholesterol (g/l)**	**Cholesterol oxidase activity (U/OD**_**600**_**)**
1	0.05	6	0.2	76	6	15	3.5	82.388
2	3.5	7	0.2	58	7	45	3.5	56.522
3	6.95	6	0.2	50	8	15	3.5	45.026
4	3.5	7	0.2	58	7	45	3.5	56.522
5	0.05	8	0.2	64	6	60	3.5	60.354
6	3.5	6	0.35	54	7	15	6.95	58.438
7	6.95	8	0.2	49	8	60	3.5	53.648
8	3.5	6	0.05	72	7	15	0.05	68.976
9	0.05	7	0.05	80	6	45	0.05	87.5612
10	0.05	7	0.35	58	6	45	6.95	63.228
11	6.95	7	0.05	53	8	45	0.05	57.48
12	6.95	7	0.35	47	8	45	6.95	41.8646
13	3.5	8	0.05	62	7	60	0.05	67.8264
14	3.5	8	0.35	51	7	60	6.95	51.732
15	3.5	7	0.2	59	7	45	3.5	55.564

**Fig. 4:**
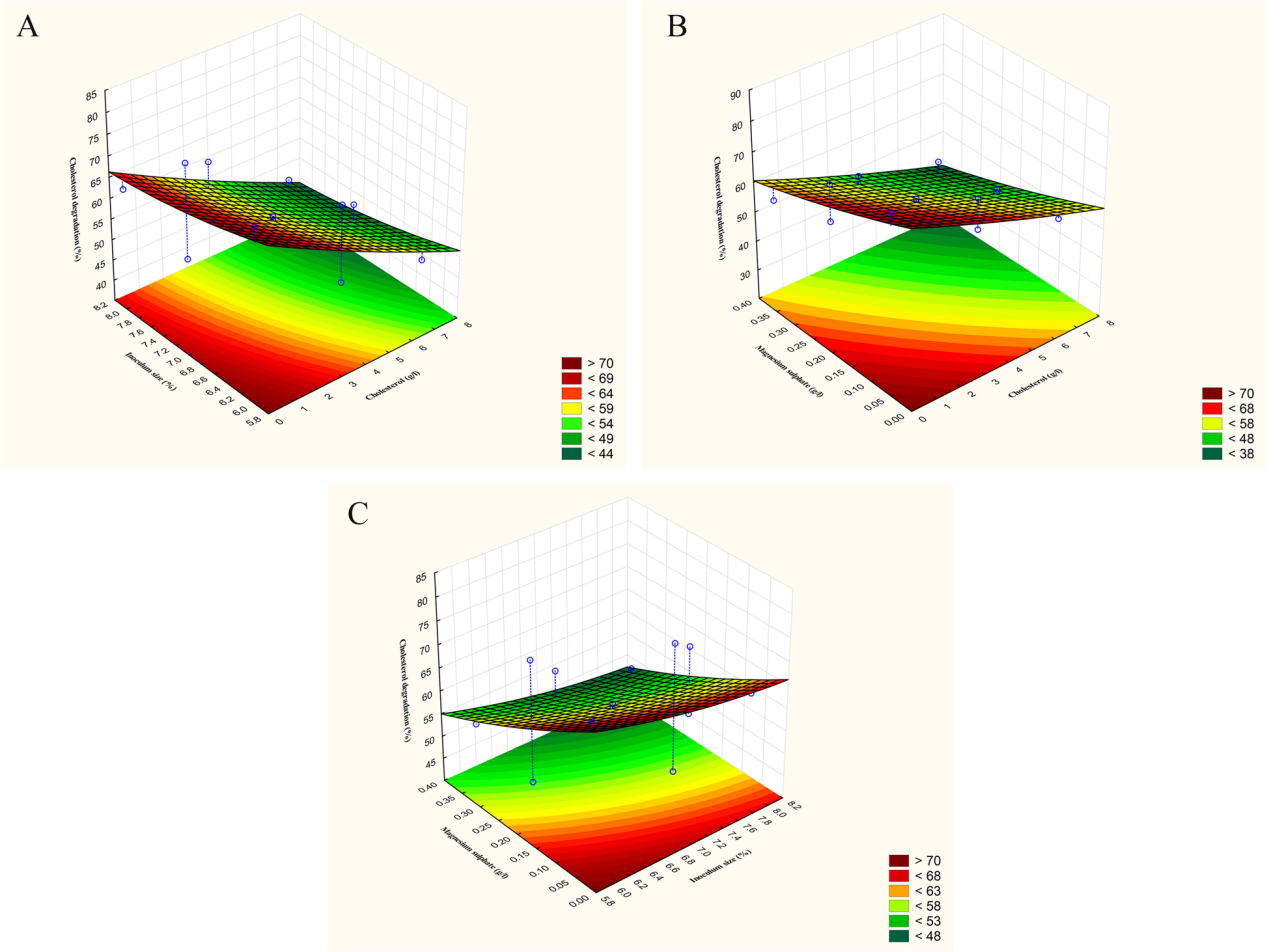
Surface and contour plots showing the interaction between cholesterol degradation (%) by *B. subtilis* versus **A** cholesterol (g/l) and inoculum size (%), **B** cholesterol (g/l) and magnesium sulphate (g/l), and **C** inoculum size (%) and magnesium sulphate (g/l)

**Fig. 5:**
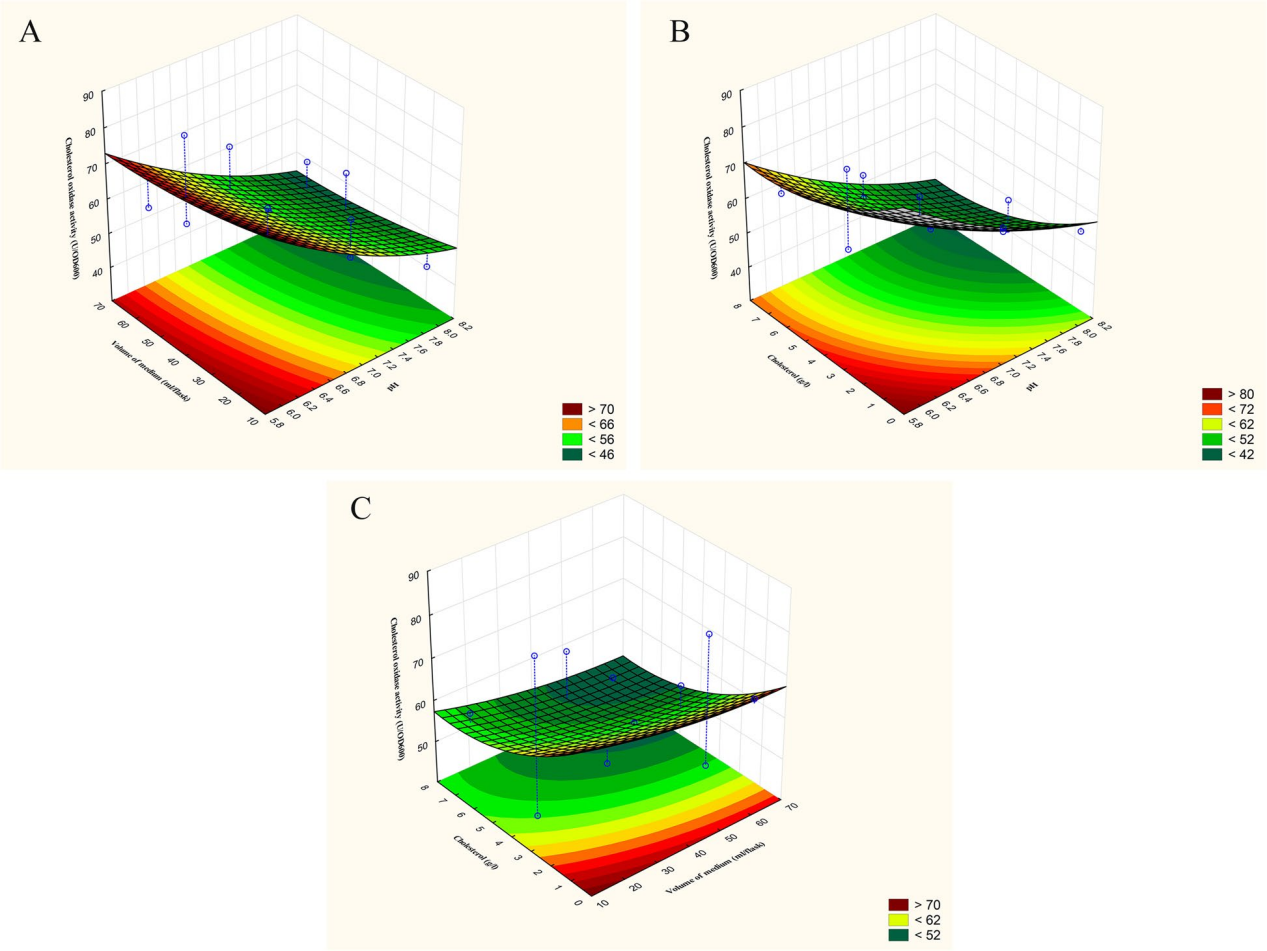
Surface and contour plots showing the relationships between *B. subtilis* cholesterol oxidase activity (U/OD_600_) versus **A** volume of the fermentation medium (ml/flask) and initial pH, **B** cholesterol (g/l) and initial pH, and **C** cholesterol (g/l) and volume of the fermentation medium (ml/flask)

According to the results stated in Table 3, the interactions of the three examined independent variables are introduced in the form of 3-D surface plots in Figs. 4 and 5. It is evident that the interactive effect of high inoculum size (%) and low cholesterol concentration (g/l) has a significant positive effect on cholesterol degradation (%) by *B. subtilis*. Furthermore, cholesterol degradation (%) improves with the decrease in the concentration of both cholesterol (g/l) and magnesium sulphate (g/l). Further decrease in the inoculum size (%) or increase in both cholesterol (g/l) and magnesium sulphate concentrations (g/l) causes a decline in the degradation of cholesterol (%). On the other hand, the activity of *B. subtilis* cholesterol oxidase (U/OD_600_) was improved by the simultaneous decrease in the volume of fermentation medium (ml/flask) and cholesterol concentration (g/l) at initial pH 6.0 till it reaches its optimum and decreases sharply on further increase.

**Fig. 6:**
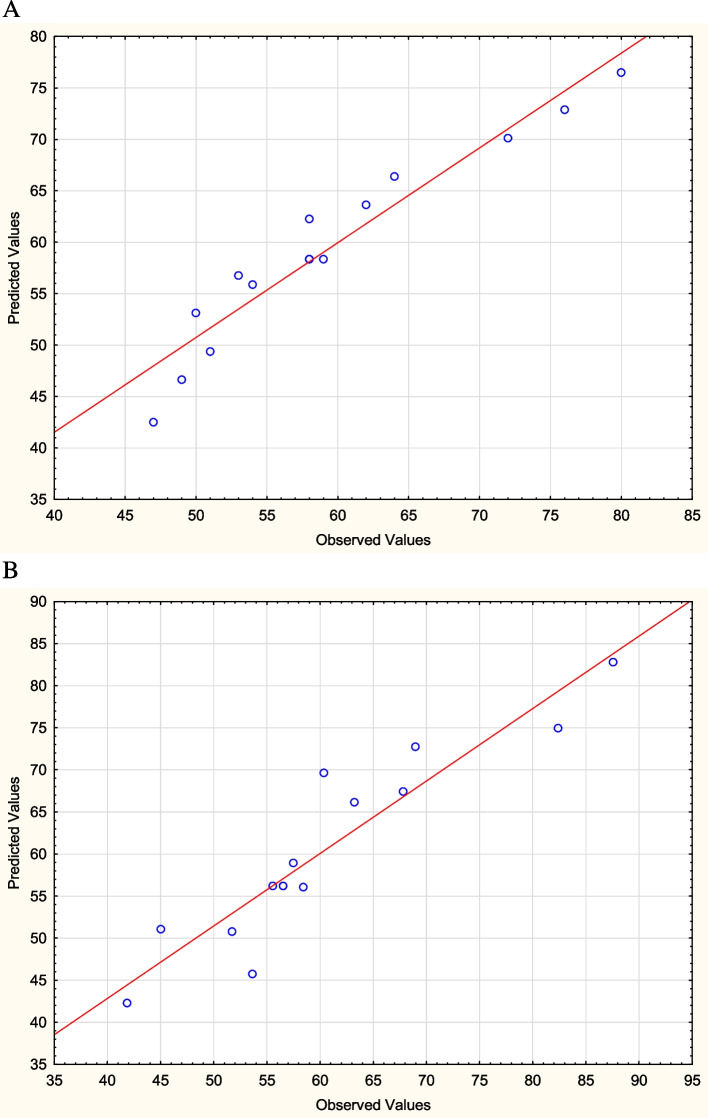
Distribution of experimental values of **A** cholesterol degradation (%) and **B** cholesterol oxidase activity (U/OD_600_) of *B. subtilis* versus predicted values (*r* = 0.99 and 0.987, respectively)

The values of coefficients and the statistical analysis are presented in Table 4. The linear effect of cholesterol concentration (*p* = 0.000064), inoculum size (*p* = 0.037113), and magnesium sulphate concentration (*p* = 0.000591) indicates their significance in cholesterol degradation (%) by *B. subtilis*. Whereas, the linear effect of fermentation medium initial pH (*p* = 0.000535) and cholesterol concentration (*p* = 0.004703) suggests their magnitude in cholesterol oxidase activity (U/OD_600_) of *B. subtilis*. The determination coefficient for cholesterol degradation (%) and cholesterol oxidase activity (U/OD_600_) are 0.9986 and 0.9842, respectively. Furthermore, as revealed by the parity plot (Fig. 6), the distribution of predicted versus experimental values reflects a linear relationship and a reasonable fitting model.

**Table 4:** Statistical analysis of the Box–Behnken experimental results

Effect	Cholesterol degradation (%)				Cholesterol oxidase activity (U/OD_600_)			
	**Parameter coefficient**	**Standard error**	**Computed *t*-value**	***p*-value**	**Parameter coefficient**	**Standard error**	**Computed *t*-value**	***p*-value**
**Intercept**	59.66667	1.062704	56.14607	0.000000	61.8389	1.769442	34.94825	0.000000
***X***_**1**_	− 9.87500	1.301542	− 7.58716	0.000064*	− 11.9391	2.147324	− 5.55998	0.000535*
***X***_**1**_^**2**^	− 0.29167	0.957909	− 0.30448	0.768530	− 1.2354	1.580387	− 0.78172	0.456889
***X***_**2**_	− 3.25000	1.301542	− 2.49704	0.037113*	− 2.6584	2.147324	− 1.23803	0.250803
***X***_**2**_^**2**^	− 0.41667	0.957909	− 0.43498	0.675076	− 0.3972	1.620404	− 0.24511	0.812545
***X***_**3**_	− 7.12500	1.301542	− 5.47428	0.000591*	− 8.3226	2.147324	− 3.87581	0.004703*
***X***_**3**_^**2**^	− 0.29167	0.957909	− 0.30448	0.768530	− 1.9300	1.580387	− 1.22120	0.256776

^*^Significant (*p*-value ≤ 0.05)

For cholesterol degradation (%): *X*_1_, *X*_2_, and *X*_3_ are the cholesterol concentration (g/l), the inoculum size (%), and the concentration of magnesium sulphate (g/l), respectively

For the activity cholesterol oxidase (U/OD_600_): *X*_1_, *X*_2_, and *X*_3_ are pH, volume of the medium (ml/flask) and cholesterol concentration (g/l), respectively

**Fig. 7:**
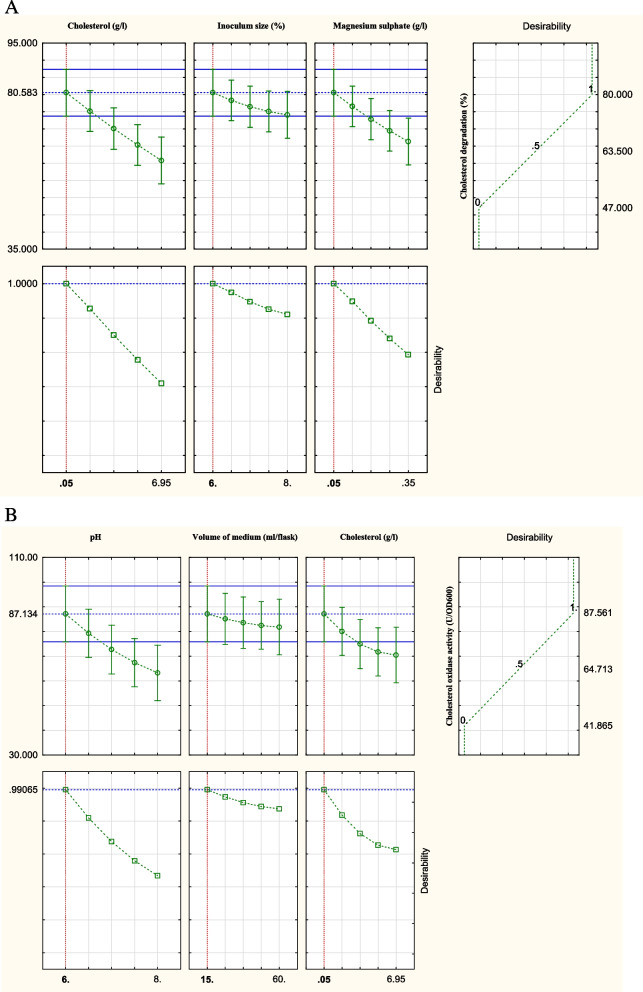
Desirability charts of variables for **A** maximum cholesterol degradation (%) by *B. subtilis* and **B** maximum cholesterol oxidase activity (U/OD_600_) of *B. subtilis* based on the results of Box–Behnken experiment

In order to predict the optimal levels for both cholesterol degradation (%) and the activity of cholesterol oxidase (U/OD_600_) of *B. subtilis*, the following two second-order polynomial functions were fitted based on the results obtained from the applied Box–Behnken experiment:

$$\mathrm{Y }= 59.67 - 9.87\mathrm{X}1 - 3.25\mathrm{X}2 - 7.125\mathrm{X}3 - 0.292\mathrm{X}{1}^{2} - 0.417\mathrm{X}{2}^{2} - 0.292\mathrm{X}{3}^{2}$$

where *Y* is the cholesterol degradation (%); *X*1, *X*2, and *X*3 are cholesterol concentration (g/l), inoculum size (%), and concentration of magnesium sulphate (g/l), respectively.

$$\mathrm{Y }= 61.84 - 11.939\mathrm{X}1 - 2.66\mathrm{X}2 - 8.32\mathrm{X}3 - 1.235\mathrm{X}{1}^{2} - 0.397\mathrm{X}{2}^{2} - 1.93\mathrm{X}{3}^{2}$$

where *Y* is the activity cholesterol oxidase (U/OD_600_); *X*1, *X*2, and *X*3 are initial pH of the fermentation medium, volume of the fermentation medium (ml/flask), and concentration of cholesterol (g/l), respectively. According to the previous equations and the desirability chart shown in Fig. 7, the predicted concentrations of cholesterol, inoculum size, and magnesium sulphate in the recommended optimal model of maximum cholesterol degradation (%) were 0.05 g/l, 6%, and 0.05 g/l, respectively. On the other hand, optimal model parametric settings of maximum cholesterol oxidase activity (U/OD_600_) were determined at initial pH of fermentation medium, 6; volume of the fermentation medium, 15 ml/flask; and concentration of cholesterol, 0.05 g/l.

**Fig. 8:**
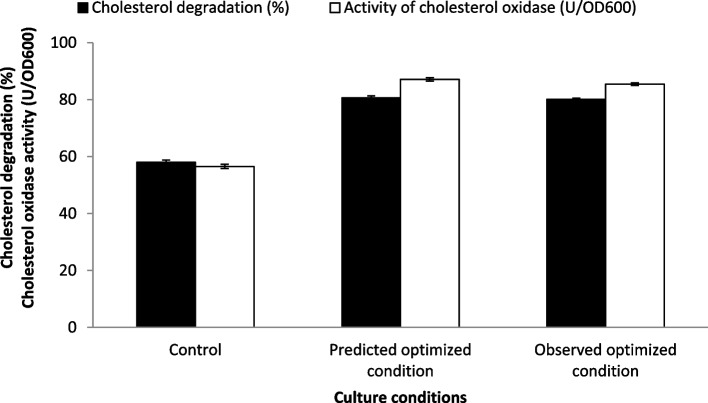
Cholesterol degradation (%) and cholesterol oxidase activity (U/OD_600_) of *B. subtilis* grown under control and optimized fermentation conditions compared to the predicted optimum conditions

For verification, a model validation experiment was subsequently performed under the predicted parameters settings for cholesterol degradation (%) and cholesterol oxidase activity (U/OD_600_). The culture condition after applying the Plackett–Burman experiment that preceded the Box–Behnken experiment was used as a control against the predicted optimized condition of the Box–Behnken experiment. As shown in Fig. 8, these optimum conditions respectively raised the cholesterol degradation (80.152%) and the activity of cholesterol oxidase (85.461 U/OD_600_) by 1.39 and 1.54 fold when compared to the control settings. Moreover, the predicted and observed values for both cholesterol degradation (%) and the activity of cholesterol oxidase (U/OD_600_) were very close.

### Gas chromatography-mass spectroscopy analysis of degradation products

**Fig. 9:**
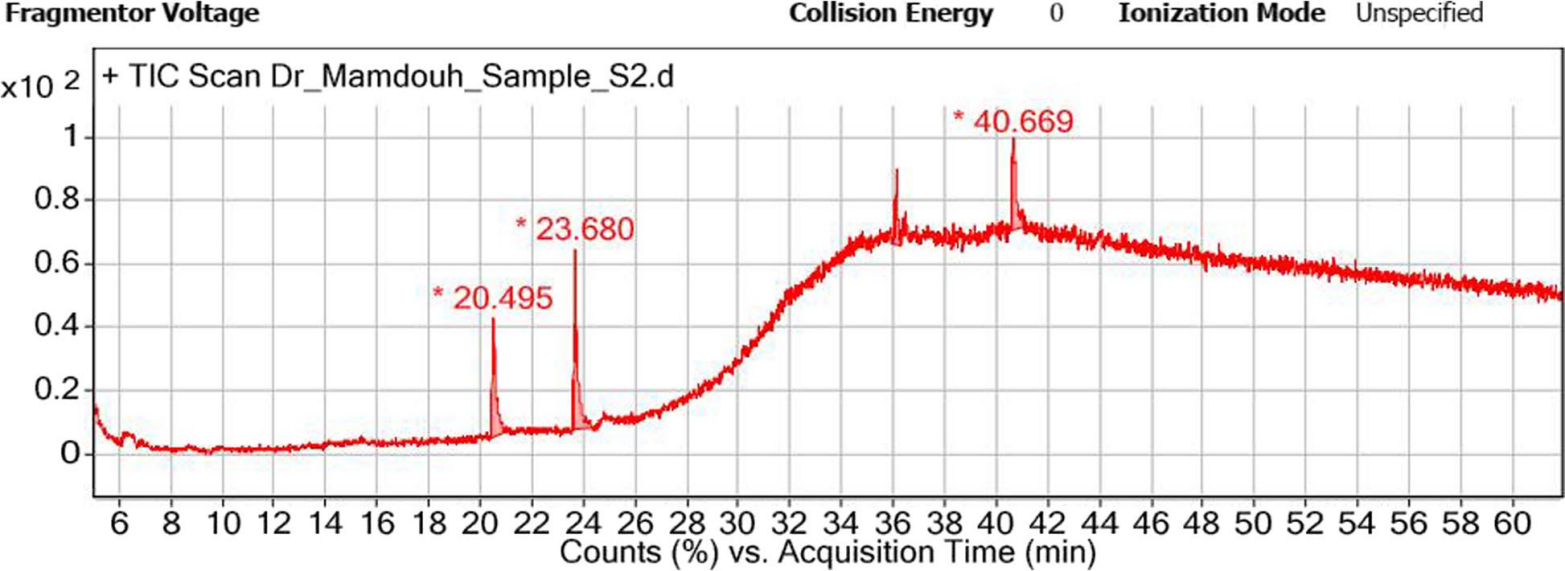
GC–MS chromatogram of the optimized fermentation medium after growth of *B. subtilis*

GC–MS was used to identify the degradation products formed in the culture medium following the growth of *B. subtilis* under optimized conditions (Fig. 9). At the end of the fermentation process, the result showed that three major end products were produced viz., 13, 16-octadecadiynoic acid, 9-octadecenoic acid, and ursodeoxycholic acid in addition to the peak of cholest-4-en-3-one which appeared at 36 min; however, no peak was detected for cholesterol.

## Discussion

Cholesterol (3β-hydroxy-5-cholestene) is a major component of all biological membranes, and it is an important precursor in the biosynthesis of other lipids. Intracellular accumulation of cholesterol is linked to high blood pressure, diabetes, peripheral vascular disease, coronary heart disease, and stroke [35–37]. Cholesterol oxidase is used for cholesterol analysis in serum samples and also has various industrial applications [2, 3]. Several bacterial species have been reported to be capable of producing cholesterol oxidase [21]. The first step of the current work was to isolate a bacterial strain from sewage that was capable of degrading cholesterol and producing cholesterol oxidase. Wali et al. [21] and Saranya et al. [38] isolated cholesterol-degrading bacterial strains from industrial wastes. The most potent strain, in terms of highest activities of cholesterol degradation (%) and cholesterol oxidase (U/OD_600_), was identified as *B. subtilis* based on the molecular sequence of the 16S rRNA gene. Few species of the genus *Bacillus* are reported to have competent ability of cholesterol degradation [15–22]. Wali et al. [21] reported that *B. Pumilus* W8 was capable of reducing cholesterol by 84% under optimized condition; however, they used the conventional OVAT optimization approach in their study. Saranya et al. [38] reported 65–80% cholesterol degradation by *Bacillus* sp. despite no optimization strategies were performed to maximize the enzyme activity. Kumari and Kanwar [16] successfully purified cholesterol oxidase from *Bacillus* sp. isolated from tiger excreta. They studied the enzyme characteristics; however, they reported no attempts to optimize the enzyme activity.

The time course of *B. subtilis* growth revealed that the maximum cholesterol degradation (%) and cholesterol oxidase activity (U/OD_600_) were attained after 32 h of incubation. This is most likely due to the availability of sufficient nutrients during the exponential growth phase. The maximum cholesterol degradation by *B. cereus* was recorded after 4 days [21] and after 24 h of incubation [17], while the maximum cholesterol oxidase activity (1.20 U/ml) was achieved by *B. cytotoxicus* after 36 h of incubation [22]. Kuppusamy and Kumar [17] reported that the optimum incubation time of *B. cereus* for the highest cholesterol oxidase activity was 32 h. *Brevibacterium sterolicum* [39], *Arthrobacter simplex* [40], *Rhodococcus* sp. [9], *Streptomyces* sp. [41], and *Enterococcus hirae* [42] have been reported to produce their maximal cholesterol oxidase activity after 24, 96, 72, 100, and 40 h of incubation, respectively.

FFD and RSM have been applied in several biotechnological applications [23–28] as they provide quick screening and evaluation of the optimal levels of variables. In the current work, according to the Plackett–Burman experiment, cholesterol concentration (g/l), inoculum size (%), and magnesium sulphate (g/l) were the most significant factors affecting cholesterol degradation (%) by *B. subtilis*. Whereas, initial pH of fermentation medium, volume of fermentation medium (ml/flask), and cholesterol concentration (g/l) were found to be significant in terms of cholesterol oxidase activity (U/OD_600_) of *B. subtilis*. The combination of medium constituents has a reflective impact on the metabolic pathways of the producer strains as it regulates the production of different metabolites [43]. Various carbon and nitrogen sources have been reported to be substrates for an enhanced cholesterol oxidase production [11]. In this context, the best nitrogen source for cholesterol degradation by *B. cytotoxicus* was the inorganic nitrogenous compounds [22]. Ahmad and Goswami [44] reported that the activity of cholesterol oxidase produced by *Rhodococcus* sp. NCIM 2891 recorded a 9.7-fold increase after applying Plackett–Burman optimization. Lee et al. [45] also investigated the effect of different carbon and nitrogen sources on the production of cholesterol oxidase by *Rhodococcus* equi no. 23. They found that cholesterol (0.1%), yeast extract (0.4–0.5%, w/v), NH_4_Cl (0.1%, w/v), NaCl (0.2%, w/v), and tween 80 (1.0%, v/v) enhanced the production of the eznyme.

Box–Behnken optimization was applied to investigate the interaction between the most significant factors affecting cholesterol degradation (%) and the activity of cholesterol oxidase (U/OD_600_) of *B. subtilis*. The Box–Behnken model revealed that 80.583% cholesterol degradation could be achieved by adjusting the concentrations of cholesterol, inoculum size, and magnesium sulphate at 0.05 g/l, 6%, and 0.05 g/l, respectively. On the other hand, the polynomial model estimated cholesterol oxidase value of 87.134 U/OD_600_ at initial pH, 6; volume of the fermentation medium, 15 ml/flask; and concentration of cholesterol, 0.05 g/l. The attained RSM optimization results are in good consistency with other reports describing RSM optimization for cholesterol oxidase production. Applying RSM approach for optimization of cholesterol oxidase of *S. lavendulae* [46], *S. badius* [47], and *S. rochei* [48] has respectively recorded an increase in the enzyme activity by 2, 2.48, and 2.58 times their initially used fermentation media. Lee et al. [49] reported a fourfold increase in the production cholesterol oxidase (0.242 U/ml) of *Rhodococcus* equi no. 23 after applying a full factorial design and central composite design. Alam et al. [50] reported a 2.6-fold increase in the activity of cholesterol oxidase (6.56 U/ml) of *Streptomyces* sp. AN after employing Plackett–Burman and Box–Behnken designs. They also found that pH, starch, NH_4_NO_3_, and FeSO_4_.7H_2_O were the most significant factors affecting cholesterol oxidase activity.

In the current study, cholesterol was used as the sole carbon source. Both cholesterol degradation (%) and cholesterol oxidase activity (U/OD_600_) of *B. subtilis* were influenced by varying the concentration of cholesterol. It has been reported that the optimal cholesterol concentration for maximum cholesterol oxidase production differs according to the producer strain. According to Rhee et al. [18], degradation of cholesterol by *Bacillus* sp. SFF34 from fermented flatfish was achieved at concentration of 0.2 g/l. The maximum degradation of cholesterol by *B. pumilus* W1 [21] and *B. cereus* [17] was recorded at cholesterol concentration of 1.0 g/l, and on increasing the concentration of cholesterol, the degradation activity decreased significantly [21]. Similar results were reported by Ouf et al. [51] on more rapid degradation of cholesterol at low cholesterol concentrations than higher concentrations by *S*. *fradiae*.

In the current work, the highest cholesterol degradation was achieved at inoculum size of 6%. Sahu et al. [52] reported an enhanced production of cholesterol oxidase at inoculum size of 11.25%; however, further increase of inoculum size resulted in a sharp decrease in enzyme production. A maximum cholesterol decomposition of 71% was recorded by *B. cytotoxicus* at 5% inoculum size [22].

The results demonstrated that the maximum degradation of cholesterol was attained at magnesium sulphate concentration of 0.05 g/l. Although the activity of cholesterol oxidase does not require metal ions [53], magnesium sulphate [54] and sodium chloride [55] have been reported to influence its activity. Therefore, other researchers supported the use of both magnesium sulphate and sodium chloride in the fermentation medium [46]. Wali et al. [21] reported that the most appropriate metallic ion for cholesterol degradation by *B. pumilus* W8 was found to be magnesium sulphate followed by calcium chloride. Similar results were reported by Ouf et al. [51] and Kuppusamy and Kumar [17] on *S*. *fradiae* and *B. cereus*, respectively.

The initial pH of the fermentation medium plays a critical role in the production of cholesterol oxidase through the submerged fermentation process as it may affect the dissociation of the medium constituents [56] and the physiological performance of cells [42]. El-Naggar et al. [57] demonstrated that the initial pH was the most significant factor affecting the production of cholesterol oxidase by *S. cavourensis* NEAE-42 strain. In the current study, the optimal initial pH of fermentation medium required for attaining the highest activity (U/OD_600_) of *B. subtilis* cholesterol oxidase was pH 6. This is in close agreement with other reports, the maximum cholesterol degradation by *B. pumilus* [21] and *Bacillus* sp. [38, 58] was attained at pH 7.0. Moreover, initial pH of the value 6.5, 7.5, 7, and 7.5 are the optimum pH for cholesterol oxidase production by *Bacillus* sp. [38, 58], *B. cereus* [17],* S. rimosus* [59] and *S. aegyptia* [32, 60], and *B. cytotoxicus* [22], respectively.

In the current study, the determination coefficient values of cholesterol degradation (%) and cholesterol oxidase activity (U/OD_600_) respectively indicated 99.86% and 98.42% match between the observed and predicted data. A regression model with a value of *R*^2^ greater than 0.9 is usually considered to have a very high correlation, and a model with a value of *R*^2^ between 0.7 and 0.9 is considered to have a high correlation [61]. Generally, optimization of *Bacillus* cholesterol oxidase has been optimized in very few studies. The cholesterol oxidase activity obtained after optimization (85.461 U/OD_600_) was compared with other previously conducted researches on various bacterial isolates. The cholesterol oxidase production by *B. pumilus* [15] and *B. cytotoxicus* [22] was optimized and their highest activities were 9 U/ml and 2.31U/ml, respectively. Similarly, the maximum reported activities of cholesterol oxidase were 6 U/ml and 20 U/mg from *Streptomyces* sp. [56, 62], 4.2 U/ml from *S. olivaceus* [52], 2.21 U/ml from *S. lavendulae* [46], 1.4 U/ml from *S. badius* [47], 5.41 U/ml from *S. rimosus* [59], 25.5 U/ml from *S. rochei* NAM-19 [48], and 4.51U/ml from *S. anulatus* NEAE-94 [63]. Likewise, the maximum activity of cholesterol oxidase produced by *Bordetella* after optimization was 1.7 U/ml [64]. Moreover, *Enterobacter* [65], *Brevibacterium* sp. [66], and *Rhodococcus* [67] showed cholesterol oxidase activity of 0.43 U/ml, 1.469 U/ml, and 0.398 U/ml, respectively.

Based on the GC–MS result, no cholesterol peak was detected at the end of the submerged fermentation that was carried out under optimized conditions indicating complete degradation of cholesterol by *B. subtilis*. GC–MS is the most commonly used method for the quantification of cholesterol [68, 69]. Similarly, Rhee et al. [18] used GC analysis to confirm the conversion of cholesterol into 4-cholesten-3-one by two novel cholesterol oxidases of *Bacillus* sp. isolated from fermented flatfish. Cholesterol oxidase catalyzes three chemical conversions: the first one is the dehydrogenation of cholesterol to produce the intermediate 5-cholestane-3-one [70]. In the second chemical conversion, the reduced flavin reacts with dioxygen yielding hydrogen peroxide and forming the oxidized enzyme state [70]. The final catalytic step is the isomerization of the double bond in the oxidized steroid ring system to form cholest-4-en-3-one [70].

## Conclusion

Cholesterol oxidase has various biomedical, environmental, and industrial applications. Optimization of the process parameters influencing the production of cholesterol oxidase by *B. subtilis* was thoroughly investigated. The Plackett–Burman optimization design was applied to search for the most significant factors affecting cholesterol degradation (%) and the activity of cholesterol oxidase (U/OD_600_) of *B. subtilis*. The degradation of cholesterol (%) and the activity of cholesterol oxidase (U/OD_600_) were enhanced by optimization of the fermentation medium using Box–Behnken design. The optimization process improved both cholesterol degradation (%) and the activity of cholesterol oxidase (U/OD_600_) by 139% and 154%, respectively. Overall, this work provides principal information for the development of efficient large-scale production of cholesterol oxidase by *B. subtilis* that could be used in various applications.

## Data Availability

All data generated or analyzed during this study are included in this published article.

## References

[1] Ohvo-Rekilä H, Ramstedt B, Leppimäki P, Slotte JP (2002). Cholesterol interactions with phospholipids in membranes. Prog Lipid Res.

[2] Kumari L, Kanwar SS (2012). Cholesterol oxidase and its applications.

[3] Pollegioni L, Piubelli L, Molla G (2009). Cholesterol oxidase: biotechnological applications. FEBS.

[4] Gadda G, Wels G, Pollegioni L, Zucchelli S, Ambrosius D, Pilone MS, Ghisla S (1997). Characterization of cholesterol oxidase from *Streptomyces hygroscopicus* and *Brevibacterium sterolicum*. Eur J Biochem.

[5] Doukyu N, Aono R (1998). Purification of extracellular cholesterol oxidase with high activity in the presence of organic solvents from Pseudomonas sp. strain ST-200. Appl Environ Microbiol.

[6] Yao K, Wang F-Q, Zhang H-C, Wei D-Z (2013). Identification and engineering of cholesterol oxidases involved in the initial step of sterols catabolism in *Mycobacterium neoaurum*. Metabol Engineer.

[7] Cheetham PS, Dunnill P, Lilly M (1982). The characterization and interconversion of three forms of cholesterol oxidase extracted from *Nocardia rhodochrous*. Biochem J.

[8] Wilmanska D, Sedlaczek L (1988) The kinetics of biosynthesis and some properties of an extracellular cholesterol oxidase produced by *Arthrobacter* sp. IM 79. Acta Microbiol Pol 37(1):45–51

[9] Watanabe K, Shimizu H, Aihara H, Nakamura R, Suzuki K-I, Komagata K (1986). Isolation and identification of cholesterol-degrading *Rhodococcus* strains from food of animal origin and their cholesterol oxidase activities. J Gener Appl Microbiol.

[10] Aunpad R (2002). Crystallization and preliminary X-ray crystallographic studies on the class II cholesterol oxidase from *Burkholderia*
*cepacia* containing bound flavin. Acta Crystallogr D Biol Crystallogr.

[11] Varma R, Nene S (2003). Biosynthesis of cholesterol oxidase by *Streptomyces lavendulae* NCIM 2421. Enz Microb Technol.

[12] Srisawasdi P, Chaichanajarernkul U, Teerakranjana N, Kroll MH (2008). Implementation of cellulomonas cholesterol oxidase for total serum cholesterol determination by the endpoint method. J Clin Lab Anal.

[13] Doukyu N, Shibata K, Ogino H, Sagermann M (2008). Purification and characterization of Chromobacterium sp. DS-1 cholesterol oxidase with thermal, organic solvent, and detergent tolerance. Appl Microbiol Biotechnol.

[14] Takagi M, Yoshida T, Taguchi H (1982). Effect of oleic acid adsorption onto cell surface on cholesterol oxidase production by *Schizophyllum commune*. J Ferment Technol.

[15] ElBaz FN, Gamal RF, ElBaz AF, Ibrahim NE, ElMekawy A (2017). Biochemical and biotechnological studies on a novel purified Bacillus cholesterol oxidase tolerant to solvent and thermal stress. Biocatal Biotransform.

[16] Kumari L, Kanwar SS (2016). Purification and characterization of an extracellular cholesterol oxidase of *Bacillus subtilis* isolated from Tiger excreta. Appl Biochem Biotechnol.

[17] Kuppusamy A, Kumar KV (2016). Optimization of cholesterol oxidase production and 16S rRNA partial sequence of *Bacillus cereus* strain KAVK4 isolated from butter. J Appl Pharmaceut Sci.

[18] Rhee C-H, Kim K-P, Park H-D (2002). Two novel extracellular cholesterol oxidases of Bacillus sp. isolated from fermented flatfish. Biotechnol Lett.

[19] Shao M (2015). Bioconversion of cholesterol to 4-cholesten-3-one by recombinant *Bacillus subtilis* expressing choM gene encoding cholesterol oxidase from *Mycobacterium neoaurum* JC-12. J Chem Technol Biotechnol.

[20] Vasanthakumar K, Kuppusamy A (2022). Enzymatic production of 4-cholesten-3-one using cholesterol oxidase from *Bacillus cereus* strain KAVK5 and its potentiality toward the inhibition of disease-associated proteins an in silico approach. J Appl Pharmaceut Sci.

[21] Wali H, Rehman FU, Umar A, Ahmed S (2019). Cholesterol degradation and production of extracellular cholesterol oxidase from *Bacillus pumilus* W1 and *Serratia marcescens* W8. BioMed Res Int.

[22] Youssef GA, El-Maghraby W, El-Aassar S (2022). Statistical optimization of fermentation conditions by Plackett-Burman methodology for a new extracellular cholesterol oxidase-producing *Bacillus cytotoxicus* strain. Egypt J Bot.

[23] Lotfy WA, Hassan SWM, Abd El-Aal AA, Ghanem KM (2019). Enhanced production of di-(2-ethylhexyl) phthalate (DEHP) by *Bacillus subtilis* AD35 using response surface methodology (RSM). Biotechnol Biotechnolog Equip.

[24] Lotfy WA, Atalla RG, Sabra WA, El-Helow ER (2018). Expression of extracellular polysaccharides and proteins by clinical isolates of *Pseudomonas aeruginosa* in response to environmental conditions. Int Microbiol.

[25] Lotfy WA, Alkersh BM, Sabry SA, Ghozlan HA (2021). Biosynthesis of silver nanoparticles by *Aspergillus terreus*: characterization, optimization, and biological activities. Front Bioengineer Biotechnol.

[26] Ghanem KM, Lotfy WA, El-Shaer MM, Elassar SA (2020). The inhibitory effect of wheat husks addition on aflatoxins production by *Aspergillus flavus* in liquid culture with various wheat compositions as carbon sources. Front Microbiol.

[27] Walid AL, Neveen MA-E-K, Ebaa EE-S, Ehab RE-H (2017). Isolation and characterization of a haloalkaliphilic protease producer bacterium from Wadi Natrun in Egypt. Afri J Biotechnol.

[28] El-Helow ER, Atalla RG, Sabra WA, Lotfy WA (2020). Kinetic studies on the expression of alginate and extracellular proteins by *Pseudomonas aeruginosa* FRD1 and PAO1. J Gener Appl Microbiol.

[29] Sambrook J, Fritsch EF, Maniatis T (1989) Molecular cloning vol 2. Cold spring harbor laboratory press, New York

[30] Brinkman FS, Leipe DD (2001) Bioinformatics: a practical guide to the analysis of genes and proteins. Wiley, New Jersey

[31] Richmond W (1973). Preparation and properties of a cholesterol oxidase from Nocardia sp. and its application to the enzymatic assay of total cholesterol in serum. Clin Chem.

[32] El-Naggar NE-A, Deraz SF, Soliman HM, El-Deeb NM, El-Shweihy, (2017). Purification, characterization and amino acid content of cholesterol oxidase produced by * Streptomyces aegyptia* NEAE 102. BMC Microbiol.

[33] Plackett RL, Burman JP (1946). The design of optimum multifactorial experiments. Biomet.

[34] Box GE, Behnken DW (1960). Some new three level designs for the study of quantitative variables. Technomet.

[35] Ordovas-Montanes JM, Ordovas JM (2012). Cholesterol, inflammasomes, and atherogenesis. Curr Cardiovasc Risk Rep.

[36] Broitman S, Cerda S, Wilkinson J (1993). Cholesterol metabolism and colon cancer. Prog Food Nutr Sci.

[37] Soliman GA (2018). Dietary cholesterol and the lack of evidence in cardiovascular disease. Nut.

[38] Saranya S, Shekinah S, Rajagopal T, Vijayakumar J, Ponmanickam P (2014). Isolation and characterization of cholesterol degrading bacteria from soap and vegetable oil industrial waste. Ind J Biotechnol.

[39] Uwajima T, Yagi H, Terada O (1974). Properties of crystalline 3 β-Hydroxysteroid oxidase of *Brevibacterium sterolicum*. Agric Biol Chem.

[40] Liu W, Hsu J, Wang W (1983). Production of cholesterol oxidase by antibiotic resistant mutant and a constitutive mutant *Arthrobacter simplex* B-7. Proc Natl Sci.

[41] Lee IA, Choe YK, Lee HS, Choe IS, Chung TW (1992). Studies on the isolation of cholesterol oxidase producing soil microorganism and the culture condition for the production of high activity cholesterol oxidase. Kor J Appl Microbiol Biotechnol.

[42] Yehia HM, Hassanein WA, Ibraheim SM (2015). Purification and characterisation of the extracellular cholesterol oxidase enzyme from *Enterococcus hirae*. BMC Microbiol.

[43] Yazdi MT, Zahraei M, Aghaepour K, Kamranpour N (2001). Purification and partial characterization of a cholesterol oxidase from *Streptomyces fradiae*. Enz Microb Technol.

[44] Ahmad S, Goswami P (2013). Enhanced production of cell-bound cholesterol oxidase from Rhodococcus sp. NCIM 2891 by the statistical method. Annals Microbiol.

[45] Lee M-T, Chen W-C, Chou C-CJB, biochemistry a, (1997). Nutritional factors that affect the production of cholesterol oxidase by Rhodococcus equi no. 23. Biotechnol Appl Biochem.

[46] Chauhan AK, Survase SA, Kishenkumar J, Annapure US (2009). Medium optimization by orthogonal array and response surface methodology for cholesterol oxidase production by *Streptomyces lavendulae* NCIM 2499. J Gener Appl Microbiol.

[47] Moradpour Z, Ghasemian A, Safari A, Mohkam M, Ghasemi Y (2013). Isolation, molecular identification and statistical optimization of culture condition for a new extracellular cholesterol oxidase-producing strain using response surface methodology. Annals Microbiol.

[48] Elsayed EA (2020). Ahmed Abdelwahed N (2020) Medium optimization by response surface methodology for improved cholesterol oxidase production by a newly isolated *Streptomyces rochei* NAM-19 strain. BioMed Res Int.

[49] Lee MT, Chen WC, Chou CCJB, biochemistry a, (1998). Maximization of cholesterol oxidase production by Rhodococcus equi no. 23 by using response surface methodology. Biotechnol Appl Biochem.

[50] Alam AA, Goda DA, Soliman NA, Abdel-Meguid DI, El-Sharouny EE, Sabry SA (2022). Production and statistical optimization of cholesterol-oxidase generated by *Streptomyces* sp. AN strain J Genet Eng Biotechnol.

[51] Ouf SA, Alsarrani AQ, Al-Adly AA, Ibrahim MK, Mohamed A-AH (2012). Evaluation of low-intensity laser radiation on stimulating the cholesterol degrading activity of Streptomyces fradiae. Part II: optimization of environmental and nutritional factors. J Taibah Univ Sci.

[52] Sahu S, Shera SS, Banik RM (2019). Optimization of process parameters for cholesterol oxidase production by MTCC 6820. Open Biotechnol J.

[53] Doukyu N (2009). Characteristics and biotechnological applications of microbial cholesterol oxidases. Appl Microbiol Biotechnol.

[54] Pathak L (2015). Artificial intelligence versus statistical modeling and optimization of cholesterol oxidase production by using *Streptomyces* sp. PLoS One.

[55] Amiri M, Najafi AA, Gheshlaghi K (2008). Response surface methodology and genetic algorithm in optimization of cement clinkering process. J Appl Sci.

[56] Praveen V, Srivastava A, Tripathi C (2011). Purification and characterization of the enzyme cholesterol oxidase from a new isolate of *Streptomyces* sp. Appl Biochem Biotechnol.

[57] El-Naggar NE-A, El-Shweihy NM, El-Ewasy SM (2016). Identification and statistical optimization of fermentation conditions for a newly isolated extracellular cholesterol oxidase-producing *Streptomyces cavourensis* strain NEAE-42. BMC Microbiol.

[58] Kokila V, Amutha K (2016). Cholesterol degrading potentiality of the bacterial isolates from Ghee. Int J Pharmaceut Sci Rev Res.

[59] Srivastava A, Singh V, Tripathi C (2018). Scale up and optimization of cholesterol oxidase production from *Streptomyces rimosus* MTCC 10792 in a 3-L bioreactor. Env Sustain.

[60] El-Naggar NE-A, Soliman HM, El-ShweihyNMJSr, (2018). Extracellular cholesterol oxidase production by * Streptomyces aegyptia*, *in vitro* anticancer activities against rhabdomyosarcoma, breast cancer cell-lines and * in vivo* apoptosis. Sci Rep.

[61] Haaland PD (2020). Experimental design in biotechnology.

[62] Niwas R, Singh V, Singh R, Tripathi D, Tripathi C (2013). Production, purification and characterization of cholesterol oxidase from a newly isolated *Streptomyces* sp. World J Microbiol Biotechnol.

[63] El-Naggar NE-A, El-Shweihy NM (2020). Identification of cholesterol-assimilating Actinomycetes strain and application of statistical modeling approaches for improvement of cholesterol oxidase production by *Streptomyces anulatus* strain NEAE-94. BMC Microbiol.

[64] Lin Y, Fu J, Song X (2010). Purification and characterization of an extracellular cholesterol oxidase from a *Bordetella* species. Proc Biochem.

[65] Kasabe PJ, Mali GT, Dandge PB (2015). Assessment of alkaline cholesterol oxidase purified from *Rhodococcus* sp. PKPD-CL for its halo tolerance, detergent and organic solvent stability. Protein Expr Purif.

[66] Yang S, Zhang H (2012). Optimization of cholesterol oxidase production by Brevibacterium sp. employing response surface methodology. Afri J Biotechnol.

[67] Ye D, Lei J, Li W, Ge F, Wu K, Xu W, Yong B (2008). Purification and characterization of extracellular cholesterol oxidase from *Enterobacter* sp. World J Microbiol Biotechnol.

[68] Pizzoferrato L, Nicoli S, Lintas C (1993). GC-MS characterization and quantification of sterols and cholesterol oxidation products. Chromatograph.

[69] Müller C, Junker J, Bracher F, Giera M (2019). A gas chromatography–mass spectrometry-based whole-cell screening assay for target identification in distal cholesterol biosynthesis. Nat Protoc.

[70] Qin H-M (2017). Refolding of a novel cholesterol oxidase from Pimelobacter simplex reveals dehydrogenation activity. Protein Expr Purif.

